# Interpreter Use and Patient Satisfaction in the Otolaryngology Outpatient Clinic

**DOI:** 10.7759/cureus.24839

**Published:** 2022-05-09

**Authors:** Hyeon Soh, Matthew L Rohlfing, Katherine R Keefe, Alexander D Valentine, Pieter J Noordzij, Christopher D Brook, Jessica Levi

**Affiliations:** 1 Otolaryngology, Boston University School of Medicine, Boston, USA; 2 Otolaryngology, University of Utah Hospital, Salt Lake City, USA; 3 Ophthalmology, University of Michigan, Ann Arbor, USA; 4 Otolaryngology - Head and Neck Surgery, Boston Medical Center, Boston, USA

**Keywords:** communication, limited english proficiency, quality improvement, interpreter, patient satisfaction

## Abstract

Background

Communication between providers and patients is essential to patient care and to the patient-physician relationship. It plays a significant role in both measurable and perceived quality of care. This study explores the satisfaction of English-speaking and limited English proficiency (LEP) patients with English-speaking providers, focusing on the correlation between patients’ primary language and the use of interpreter services on patients’ visit satisfaction.

Methodology

This study was designed to have a sample size sufficient to detect a 10% difference in the primary outcome, overall visit satisfaction, between language-concordant patients and LEP patients in the interpreter and no interpreter groups, assuming a two-tailed alpha of 0.05 and power of 80%. All collected data were analyzed using the Statistical Package for the Social Sciences software, version 25 (IBM Corp, Armonk, NY, USA), and significance was determined if p <0.05.

Results

Of the total 209 patients, 65 utilized professional interpreter services, nine used an ad-hoc interpreter, and 135 did not require an interpreter. Patients who used an interpreter demonstrated lower visit satisfaction compared with patients who did not (p < 0.001). Patients expressed significantly greater preference for in-person interpreter (mean = 9.73) or a family member (mean = 9.44) compared to telephone services (mean = 8.50) (p = 0.002). The overall satisfaction scores did not significantly differ between different interpreter types (p = 0.157).

Conclusions

LEP patients experienced lower visit satisfaction compared to language-concordant patients. The data suggest that perceived quality of communication was a factor in these lower satisfaction reports. While LEP patients did prefer in-person interpreters, there was no significant difference in overall visit satisfaction between different types of interpreters.

## Introduction

Studies have shown that patients with limited English proficiency (LEP) face a significantly greater number of adverse events, have less trust in the quality of care, and have a lower likelihood of treatment adherence and continued follow-up. They also often have more limited access to healthcare [1-5]. In 2013, approximately 61.6 million individuals in the United States spoke a language other than English at home, and 25.1 million also had LEP. Overall, in 2013, the LEP population represented 8% of the total US population aged five and older [6]. These numbers continue to rise, making care for this population increasingly important [7].

Professional interpreters have been shown to improve overall care for LEP patients, including contributing to improved comprehension, clinical outcomes, and better satisfaction with the care provided [3,8-11]. There are several ways to provide interpretation for LEP patients. Professional interpreters include in-person, phone, or video services. In some situations, untrained or ad-hoc interpreters are used, which may include family, friends, or clinic staff. These methods have benefits and limitations [3,4,10,12-14]. In-person interpreters are able to see a patient’s body language or emotional cues, but providing such an interpreter for all visits can be challenging and costly. Phone and video services offer a cost-effective alternative to expanding interpreting services, but there are inherent difficulties that may limit the effectiveness of communication and cause frustration for both patients and providers [3,12,14]. Patients often bring family or friends who are willing to interpret, but these individuals are not professionally trained and may not be fully faithful and accurate [7,13]. Interpreters also contribute important cultural understanding and provide benefits beyond simple word-for-word translation [6]. To our knowledge, there is a limited number of published studies from Otolaryngology clinic settings with a primary focus on LEP patients’ satisfaction as it relates to interpreter services [15]. Moreover, existing studies from other specialties have shown mixed results when investigating patient satisfaction with interpreters. This study seeks to explore the satisfaction of English-speaking and LEP patients with English-speaking providers, focusing on the effect of the patient’s primary language and the use of interpreter services.

This article was previously presented as a poster presentation at the 2020 Combined Otolaryngology Spring Meetings on May 15, 2020.

## Materials and methods

This study was approved by the Institutional Review Board at Boston Medical Center after initially being designed as a quality improvement project. A total of 209 surveys were collected from June 14th, 2018, to July 3rd, 2019, and the data were analyzed retrospectively. The study was designed to have a sample size sufficient to detect a 10% difference in the primary outcome, overall visit satisfaction, between language-concordant patients and LEP patients in the interpreter and no interpreter groups, assuming a two-tailed alpha of 0.05 and power of 80%. Because the data were collected for quality improvement, the total eligible population and enrollment goal were not determined prospectively.

The study was conducted in an outpatient otolaryngology clinic at an urban, safety-net, tertiary-care, academic medical center with a robust trained medical interpreter service program. All LEP patients were offered the use of an interpreter, and in-person interpreters were utilized when possible. If an in-person interpreter was not readily available, a phone or video interpreter was utilized instead. Some patients declined interpreter services and preferred that a family member assists in communication. In these cases, the family members were allowed to interpret, but any surgical consent discussions or discussions involving critical decision-making were conducted with a trained interpreter. Each appointment was conducted according to the physicians’ typical protocols, without regard to the survey collection. After the visit, the patients were greeted by a clinical assistant who offered the opportunity to complete the satisfaction survey. The surveys were offered in English, Spanish, or Haitian Creole. No specific assistance was provided because the surveys had language-concordant, written instructions. Patients whose visits were conducted in other languages and those under 18 years old were not offered to participate.

The survey was modified from the Press Ganey surveys, which are widely used as a patient satisfaction metric. The survey included questions related to wait time and wait time satisfaction, physician communication, physician rating, and rating of the clinic. The survey also included questions regarding interpreter use, type and mode of interpreter, and satisfaction with interpreter services. The survey was anonymous and did not contain any patient identifying information.

The main outcome variable was the “overall visit satisfaction score.” Maximal rating from nine survey questions was collected to calculate the overall visit satisfaction score. Secondary outcomes of interest were satisfaction with the interpreter (only completed by LEP patients) and the patient’s rating of their doctor on a scale of one to ten (this was also included in the total survey score).

Interpreter and no interpreter groups were compared based on visit-related variables. Two-tailed, independent sample t-tests and one-way analysis of variance (ANOVA) were used to compare the means for the primary outcome with binary and non-binary outcomes, respectively. Confidence intervals (CIs) were calculated for all reported means. The Chi-square test or Fisher’s exact test (when an expected count was less than five) was used to compare distributions for categorical variables. A linear regression model for the total survey score was generated using potential covariates identified from univariate analyses. Covariates were selected for inclusion in the regression model when there was a significant difference between comparison groups or when they demonstrated a significant relationship with the total survey score in univariate analysis. All collected data were analyzed using the Statistical Package for the Social Sciences software, version 25 (IBM Corp, Armonk, NY, USA), and significance was determined if p <0.05 [16].

## Results

A total of 209 patients completed the survey and were included in the final analysis, of whom 65 utilized interpreter services, nine used an ad-hoc interpreter, and 135 did not require an interpreter. Of the 209 patients, Attending 1 conducted clinic visits with 119 patients: 32 (26.9%) visits with interpreter services and 87 (73.1%) without; and Attending 2 conducted clinic visits with 90 patients: 42 (46.7%) visits with interpreter services and 48 (53.3%) without. The demographic data are presented in Table 1.

**Table 1:** Demographic variables and covariates stratified by the participation of interpreters in clinic visits.

Variable	Total (N = 209)		Interpreter (N = 74)		No interpreter (N = 135)		P-value
	No.	%	No.	%	No.	%	
Attending provider							
Attending #1	119	56.9%	32	43.2%	87	64.4%	0.004
Attending #2	90	43.1%	42	56.8%	48	35.6%	
Resident							
Yes	45	21.5%	12	16.2%	33	24.4%	0.218
No	164	78.5%	62	83.8%	102	75.6%	
Non-physician provider							
Yes	12	5.7%	7	9.5%	5	3.7%	0.119
No	197	94.3%	67	90.5%	130	96.3%	
Medical student							
Yes	67	32.1%	15	20.3%	52	38.5%	0.008
No	142	67.9%	59	79.7%	83	61.5%	
Scribe							
Yes	102	48.8%	17	23.0%	85	63.0%	<0.001
No	107	51.2%	57	77.0%	50	37.0%	
Wait time							
<15 minutes	136	65.1%	48	64.9%	88	65.2%	0.624
15–30 minutes	49	23.4%	20	27.0%	29	21.5%	
30–45 minutes	18	8.6%	5	6.8%	13	9.6%	
45+ minutes	6	2.9%	1	1.4%	5	3.7%	
Wait time satisfaction							
Top box	110	52.6%	38	51.4%	72	53.3%	0.885
Non-top box	99	47.4%	36	48.6%	63	46.7%	

**Figure 1:**
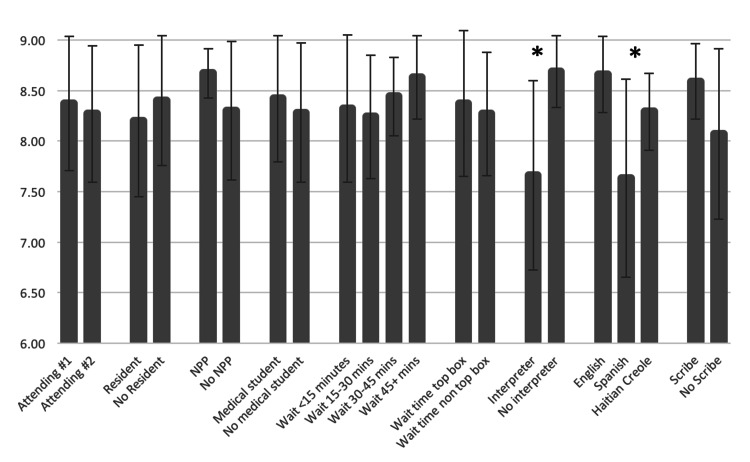
Summary of the mean survey scores for different variables associated with clinic visits. The score is a composite representing top-box responses on a modified Press Ganey survey (scale 0-9). Means that are significantly different at p < 0.05 are represented by “*”. For interpreters, p < 0.001. For language, p < 0.001. For scribes, p = 0.005. NPP = non-physician provider

LEP patients who used interpreter services reported lower visit satisfaction than language-concordant patients (means = 7.66 [95% CI = 7.32 to 8.43] and 8.69 [95% CI = 8.53 to 8.78], respectively; p < 0.001). Survey scores also differed according to patients’ primary language, with English-speaking patients having the highest mean survey scores (8.66 [95% CI = 8.53 to 8.78]), followed by Haitian Creole-speaking patients (8.29 [95% CI = 7.59 to 8.98]), followed by Spanish-speaking patients (7.63 [95% = CI 7.15 to 8.12]; p < 0.001) (Figure 1, Table 2). There was expected covariance between primary language and interpreter use (all non-English-speaking patients used interpreters).

**Table 2:** Demographic variables and covariates with the associated survey scores (0-9 scale). P-values were determined with independent-sample t-test for binary variables and ANOVA for non-binary variables. ANOVA = analysis of variance; CI = confidence interval

Variable	Overall (N = 209)	Survey total, mean	95% CI, Low	95% CI, High	Standard deviation	P-value
Attending provider						
Attending #1	119	8.37	7.98	8.55	1.33	0.582
Attending #2	90	8.27	8.13	8.61	1.35	
Resident						
Yes	45	8.20	7.75	8.65	1.50	0.479
No	164	8.40	8.16	8.56	1.29	
Non-physician provider						
Yes	12	8.67	8.35	8.98	0.49	0.364
No	197	8.30	8.11	8.50	1.37	
Medical student						
Yes	67	8.42	8.11	8.72	1.25	0.493
No	142	8.28	8.05	8.51	1.38	
Wait time						
<15 minutes	136	8.32	8.08	8.57	1.46	0.872
15–30 minutes	49	8.24	7.90	8.59	1.22	
30–45 minutes	18	8.44	8.05	8.83	0.78	
45+ minutes	6	8.63	7.81	9.52	0.82	
Wait time satisfaction						
Top box	110	8.37	8.10	8.64	1.44	0.591
Non-top box	99	8.27	8.03	8.52	1.22	
Interpreter						
Yes	74	7.66	7.23	8.09	1.87	<0.001
No	135	8.69	8.57	8.81	0.71	
Survey language						
English	137	8.66	8.53	8.78	0.75	<0.001
Spanish	65	7.63	7.15	8.12	1.96	
Haitian Creole	7	8.29	7.59	8.98	0.76	
Scribe						
Yes	102	8.59	8.44	8.74	0.75	0.005
No	107	8.07	7.75	8.40	1.69	

There were differences between interpreter and non-interpreter groups with regards to attending physician, presence of scribe, and participation of medical students. Bivariate analyses were used to select potential confounding variables, and those variables were used to construct a multivariate logistic regression model. After controlling for attending, scribe, and medical student participation, the presence of an interpreter was significantly associated with the total survey scores (coefficient = -0.96, 95% CI = -1.34 to -0.57; p < 0.001 (Table 3). Language types were not included in the multivariate analysis due to their covariance with interpreter use.

**Table 3:** Linear regression model for survey score (maximum score 9) using potential covariates identified from univariate analyses. CI = confidence interval

Variable	Coefficient	95% CI Low	95% CI High	Sig.
Attending #1	-0.26	0.79	16.32	0.26
Scribe	0.36	-0.71	0.19	0.13
Medical student	-0.10	-0.11	0.82	0.67
Interpreter	-0.96	-1.34	-0.57	<0.001
Constant	12.93	8.33	9.00	-

The wait time (p = 0.312) and attending (p = 0.480) did not show significant association with the overall visit satisfaction. Longer wait times did not have a significant association with the satisfaction score compared to shorter wait times (mean = <15 minutes = 8.32 [95% CI = 8.08 to 8.57], 15-30 minutes = 8.24 [95% CI = 7.90 to 8.59], 30-45 minutes = 8.44 [95% = CI 8.05 to 8.83], >45 minutes = 8.63 [95% CI - 7.81 to 9.52]; p = 0.624). There was no significant association between wait time and the type of interpreter services utilized (p = 0.611).

Overall, a total of nine patients reported an overall satisfaction score below 4. Of these nine patients, eight required an interpreter, and all those patients reported negative perceptions of physician communication. The close relationship between visit satisfaction and effective communication remained for the 49 Spanish-speaking patients who used interpreters and responded to questions about physician communication with top-box responses. In total, 45 (81.6%) of those patients rated satisfaction scores greater than or equal to 8. The average visit satisfaction score for Spanish-speaking patients with non-top-box responses to physician communication questions was 4.90, whereas among those with top-box responses was 8.50. The average rating of interpreter (scale 0-10) among patients who expressed difficulty communicating was 9.38, similar to those who did not express difficulty whose mean interpreter rating was 9.25.

The type of professional interpreter was equally divided between phone and live interpreters. Although patients who utilized interpreter services expressed significantly greater interpreter satisfaction with in-person interpreters (mean = 9.73, 95% CI = 9.51 to 9.95) or family members (mean = 9.44, 95% CI = 8.58 to 10.31) compared to telephone services (mean = 8.50, 95% CI = 7.82 to 9.18) (p=0.002), the overall survey satisfaction scores did not significantly differ between different types or modes of interpreter used (p = 0.157) (Table 4).

**Table 4:** Selected outcomes stratified by interpreter type. CI = confidence interval; SD = standard deviation

Variable	Phone or video interpreter (N = 32)					In-person interpreter (N = 33)					Family member (N = 9)			
	Mean	95% CI, Low	95% CI, High	SD	Mean		95% CI, Low	95% CI, High	SD	Mean	95% CI, Low	95% CI, High	SD	P-value
Interpreter rating (scale 1–10)	8.50	7.82	9.18	1.88	9.73		9.51	9.95	0.63	9.44	8.58	10.31	1.13	0.002
Survey score (scale 0–9)	7.56	6.9	8.23	1.85	7.45		6.73	8.18	2.05	8.78	8.44	9.12	0.44	0.157

## Discussion

Providing adequate interpretation services to LEP patients can be a significant challenge. These challenges are particularly prevalent in urban and safety-net hospitals with large populations of LEP patients and potentially limited resources. The results of this study showed lower visit satisfaction among LEP patients compared with patients who did not require an interpreter. The difference in survey scores was not better accounted for by covariates such as participation of medical students or scribes, wait time, or attending.

Similar findings were reported in a study conducted in a primary care and emergency department (ED) clinic in an urban New York City setting. In this study, Gany et al. found that the language-concordant group rated physicians higher than the language-discordant group regardless of the interpreter type used [5]. In particular, the study reported that both understanding physicians’ explanations of procedures and results and understanding their instructions for follow-up care were inferior for patients in the interpreted medical encounter. Similarly, a study conducted in a Boston ED reported that non-English speakers were significantly less satisfied with the visit and were significantly less likely to return to that provider’s clinic even after controlling for other confounders. In this study, the type of interpreter utilized was not clearly identified [17]. However, in a 2002 study conducted in a walk-in urgent care clinic in Denver, Lee et al. found no difference in overall satisfaction between the language-concordant group and LEP patients utilizing a telephone interpreter. In fact, the only group in this study that expressed significantly lower visit satisfaction than other groups were those who utilized family, friends, or ad-hoc clinic staff interpreters. As the authors of the study noted, such discrepancies may be due to variations in survey questions, interpreter services, and clinic type [12]. Additionally, greater variations in cultural and social backgrounds (such as cultural expectations, socioeconomic factors, or education levels) that are likely to be more prevalent in large metropolitan safety-net institutions may contribute in unexpected ways to patient satisfaction. These cultural differences are represented effectively in an anecdotal article written by a professional interpreter, where they describe the many communication barriers that exist outside of “verbatim itself” [18]. These may include difficult-to-interpret concepts, cultural or spiritual beliefs surrounding illness, and cultural attitudes toward medical providers, among others.

In this study, all LEP patients who experienced very low visit satisfaction (score 4 or less out of a possible 9) expressed that it was difficult to understand the doctor’s instructions. This was despite the use of professional interpreter services. This small group of patients actually reported full satisfaction (all 10/10 ratings) with their interpreter services. Although conclusions from this small subgroup are limited, it should be noted that the patient perception of interpreter quality was high while their perception of physician communication was low. This suggests that some communicational difficulties that result in dissatisfaction are independent of the quality or perceived quality of interpretation and may be more dependent on physician communication strategies.

Among the types of interpreter services studied, in this study, in-person interpreters and family members were rated more highly than telephone or video interpreters. The overall visit satisfaction scores, however, did not differ significantly between different interpreter types.

Similar to the results of this study, prior studies from different specialties also reported that while the in-person interpreter encounters were better perceived by patients and providers, there was no quantitative difference between in-person, video, and telephone interpretation in overall visit satisfaction despite the longer wait-times when using video interpretation [12-14]. However, a 2020 study conducted within a pediatric otolaryngology clinic in Chicago showed that families and employees reported significantly greater overall visit satisfaction with in-person and video interpreter use [15]. Similarly, in a small randomized controlled trial conducted in a well-baby clinic, Hornberger et al. reported that both physicians and mothers unanimously preferred a remote simultaneous interpretation service and that there was improved accuracy and increased discussion between physicians and the mothers [19].

The discrepancies within study outcomes may be due to several factors. To begin, individual interpreter competence, training, and the need for robust interpretation are not standardized across different hospitals for direct outcome comparison. Such issues may be more prominent in newer modes of technology, including video interpreters. Moreover, subtle differences across hospitals, types of interpretation, and clinic types could also contribute to different outcomes.

There are several limitations to this study. It was conducted in a single academic medical center with two attending providers, and the results may not be generalizable to practices of different types or practices without a robust interpreter services program. Additionally, the interpreter type used was not randomized and was self-reported on the surveys. While potential confounders were recorded and controlled for as possible, there may be unidentified confounding variables. Lastly, only two non-English languages were analyzed in this study, and there may be unidentified cultural factors that contribute to interpreter utility.

## Conclusions

This study demonstrated that LEP patients experienced lower visit satisfaction compared to language-concordant patients. While LEP patients did prefer in-person interpreters, there was no significant difference in overall visit satisfaction between different types of interpreters. A meaningful physician-patient encounter requires two-way communication, and effective interpretation can help create this bridge. Additional studies in the otolaryngology clinic setting should explore more granular factors surrounding visit experience for the LEP patient population to allow for improvements in care.
